# An overview of hypopituitarism’s causes

**DOI:** 10.3389/fendo.2025.1695833

**Published:** 2026-01-07

**Authors:** Yinbei Zhang, Zhiyue Chen, Lin Sun, Weiying Guo

**Affiliations:** Department of Endocrinology and Metabolism, The First Hospital of Jilin University, Changchun, Jilin, China

**Keywords:** hypopituitarism, endocrinology, pituitary adenoma, Sheehan syndrome, tuberculosis

## Abstract

The widespread application of tumor therapies such as immune checkpoint inhibitors and the emergence of new infectious diseases such as COVID-19 are promoting the continued expansion of the cause spectrum of hypopituitarism, making its scope significantly beyond traditional causes such as pituitary tumors and craniocerebral trauma. Faced with this evolution, a comprehensive and in-depth understanding of its etiology has become a top priority, which has also put forward new requirements for clinical diagnosis and differential diagnosis. This review aims to systematically sort out and deeply explore the etiology and pathogenesis of this disease. The content not only covers traditional factors such as pituitary tumors, radiation injury, and pituitary surgery, but also the latest progress in emerging fields such as immunotherapy, new infections, and autoimmunity. It aims to provide reliable reference for clinicians’ diagnosis and treatment practice and lay a theoretical foundation for future research in this field.

## Graphical Abstract

**Figure:**
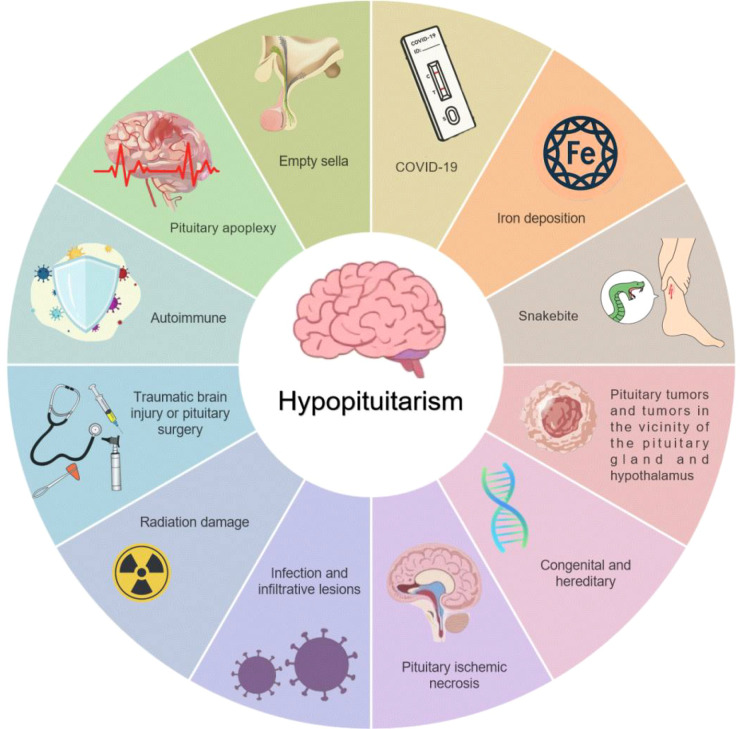

## 1 Introduction

Damage to the hypothalamus, hypothalamic-pituitary pathway, pituitary gland, or neurohypophyseal gland for various reasons leads to pituitary dysfunction, a disorder characterized by insufficient secretion of one or more pituitary hormones or diabetes insipidus. International studies indicate that the prevalence and incidence of hypopituitarism in adults are roughly 37.5–45.5 and 2.1–4.2 per 100,000 individuals annually, respectively, and the disease spectrum shows significant age and geographical heterogeneity (1). For example, in children, the incidence of the disease can be as high as 1/16,000 to 1/26,000, and the causes are mostly congenital or hereditary (2). The disease is not only complex to diagnose, but is also closely related to severe clinical outcomes. Studies have confirmed that the standardized mortality rate (SMR) of the disease is almost eight times higher than the SMR of the general population. The SMR is significantly higher in young patients and men, which highlights the importance of early identification and intervention (3). The etiological spectrum of this disease is currently undergoing profound and dynamic evolution, and emerging causes such as immunotherapy and emerging infections are constantly emerging (4). Therefore, this review aims to integrate the latest evidence and clinical insights and strive to provide endocrinologists with an etiological diagnosis framework that keeps pace with the times and has both depth and breadth to cope with increasingly complex clinical practice needs.

### 1.1 Neoplasms and masses in the sellar region

Sellar tumors and space-occupying lesions are the most common causes of acquired hypopituitarism, accounting for approximately 50% of the total cases (5). It is noteworthy that with the widespread use of sensitive MRI, pituitary incidentalomas are frequently discovered, underscoring the clinical importance of this category of lesions (6).As shown in **Table 1**, this systematic classification framework helps to deeply understand its diverse pathogenesis. Primary pituitary tumors (mainly pituitary neuroendocrine tumors) mainly compress normal pituitary tissue through mass effect, affect blood supply, and even cause pituitary apoplexy, leading to hypofunction and central diabetes insipidus (7). In addition, they are also driven by specific molecular changes, such as the dysregulation of KMT5A or SRPK1-related pathways in certain tumor cells (8). Secondly, for various primary intrasellar and parasellar tumors (such as craniopharyngioma, meningioma, and glioma), in addition to the mass effect, the chronic inflammation caused by them or the direct invasion of the hypothalamic-pituitary pathway are also important pathogenic mechanisms (9). Finally, regarding metastatic tumors, although relatively rare, breast and lung cancers constitute the main primary sites (10). The main route of metastasis is hematogenous dissemination, especially through the posterior pituitary artery system, which explains why such patients often present with central diabetes insipidus as the first manifestation (11). It is worth noting that bone metastases adjacent to the skull base can also cause disease through direct invasion of the gland. Although rare, metastases from other primary sites, such as colon cancer, have also been documented (12). Therefore, clinicians need to include pituitary metastases in the differential diagnosis of any patient with known malignancy and pituitary dysfunction.

**Table 1:** Classification of neoplasms and masses in the sellar region and their pathogenic features.

Category	Specific entities	Key pathogenic mechanisms	Characteristic clinical notes
Primary Pituitary Tumors	Pituitary neuroendocrine tumors (PitNETs)	- Mass effect (compression, ischemia, apoplexy) - Specific molecular alterations (e.g., KMT5A, SRPK1 pathways)	Most common cause of sellar mass-related hypopituitarism.
Primary Sellar/Parasellar Tumors	Craniopharyngioma, Meningioma, Glioma	- Mass effect - Chronic inflammation - Direct invasion of the hypothalamic-pituitary pathway	Often involve both anterior and posterior pituitary dysfunction.
Metastatic Tumors	- Most Common: Breast cancer, Lung cancer - Rare: Colorectal cancer, etc.	- Hematogenous dissemination (via posterior pituitary artery) - Direct invasion from skull base metastases	Central diabetes insipidus is frequently the initial manifestation.

### 1.2 Structural and vascular lesions of the hypothalamic-pituitary axis

The integrity of the hypothalamic-pituitary (HP) axis is fundamental for neuroendocrine homeostasis. Disruptions to this axis, whether from chronic structural compression or an acute vascular event, represent primary causes of acquired hypopituitarism and diabetes insipidus (13). Structural lesions, such as suprasellar tumors (such as craniopharyngioma, glioma, etc.), radiation damage (especially after extensive cranial radiotherapy), and invasive lesions (such as (lymphoma, leukemia, etc.)), usually lead to progressive and selective hormone deficiency through mass effect and secondary inflammation (14). This occurs through mechanisms such as mass effect, direct invasion, and secondary inflammation, which ultimately impair the production or transport of hypothalamic-releasing hormones. For example, A classic manifestation is tertiary adrenal insufficiency, resulting from deficient corticotropin-releasing hormone (CRH) secretion (15). In contrast, pituitary insufficiency Stroke is an acute life-threatening clinical syndrome caused by pituitary hemorrhage and/or infarction (16). Its core pathophysiological basis can be attributed to the decompensation of the internal blood supply of the pituitary tumor, which triggers ischemia and hypoxia. This microenvironment triggers a complex molecular cascade: hypoxia-inducible factor 1 (HIF-1α), as the core molecule of hypoxia sensing, is activated, which in turn upregulates vascular endothelial growth factor (VEGF) and tumor necrosis factor α (TNF-α) and other key factors (17). VEGF promotes the formation of fragile new blood vessels with abnormal structure, while TNF-α damages the integrity of blood vessels and increases permeability (17). The two synergistically increase the risk of bleeding. At the same time, cyclooxygenase-2 (COX-2), pituitary tumor transforming gene (PTTG) and fibroblast growth factor are highly expressed in aggressive tumors (18). FGF and various matrix metalloproteinases jointly shape the tumor phenotype prone to stroke by promoting local inflammation, driving tumor proliferation and angiogenesis, and degrading the vascular basement membrane (18). Therefore, whether it is acute or chronic, a comprehensive assessment of anterior and posterior pituitary function must be performed. There is no need to wait for hormone results, and stress-dose glucocorticoids should be administered immediately (19).

### 1.3 Congenital hereditary

Congenital hypopituitarism (CPHD) is a rare neuroendocrine disorder with an incidence of approximately 1/4,000 to 1/10,000 live births (20). The essence of the disease is a disorder of the embryonic development program of the pituitary gland. The normal development of the pituitary gland begins with two independent anlage: the adenohypophysis originates from the Rathke’s pouch formed by the invagination of the oral ectoderm, while the neurohypophysis and pituitary stalk originate from the neuroectoderm of the ventral diencephalon (21). This delicate process is precisely regulated by complex signaling pathways (such as FGF, BMP) and transcription factor cascades. Mutations in genes that regulate the development of the pituitary-hypothalamic region are the core cause of CPHD (22). Such mutations often result in defects in the development of midline structures, with a phenotypic spectrum ranging from severe holoprosencephaly (HPE) to the characteristic septal dysplasia (SOD). These structural abnormalities are often associated with pituitary stalk interruption syndrome (PSIS), in which the pituitary stalk is thin or absent and associated with anterior pituitary hypoplasia (23). The genetic etiology of CPHD is highly heterogeneous. HESX1 gene mutation is the classic genetic cause of SOD. In addition to CPHD, patients are often accompanied by optic nerve hypoplasia and absence of septum pellucidum (24).Haploinsufficiency of the OTX2 gene can lead to syndromic hypopituitarism, and its ocular phenotype is much more severe than dry eye disease, often including severe structural abnormalities such as anophthalmia or microphthalmia (25).In addition, CPHD is also one of the common features of complex genetic diseases such as Kallmann syndrome (26), septum pellucidum malformation (27), and Prader-Willi syndrome (28). Although the application of next-generation sequencing technologies (such as whole-exome sequencing) has greatly accelerated the discovery of disease-causing genes, about 85% of CPHD cases still cannot obtain a clear molecular diagnosis (29). Developmental biology research continues to provide important clues for the identification of candidate genes for human diseases through embryonic lethal mouse models. While these studies continue to fill gaps in our understanding of pituitary development, they also provide new directions for future genetic diagnosis (30). In view of the high clinical and genetic heterogeneity of CPHD, systematic genetic testing of suspected patients combined with multidisciplinary evaluation is crucial to achieve accurate diagnosis, prognosis and genetic counseling (31).

### 1.4 Pituitary ischemia and necrosis

Ischemic necrosis of the pituitary gland is characterized by hypoxia and tissue necrosis due to an inadequate blood supply, which ultimately results in pituitary hypofunction. The gland is particularly vulnerable to ischemia because of its reliance on a low-pressure portal venous system (32). Sheehan syndrome, diabetic vascular disease, and other conditions are common causes. Pituitary infarction from pregnancy, labor, or postpartum hemorrhage is the main cause of Sheehan syndrome (SS). During pregnancy, pituitary hypertrophy increases its metabolic demand, while the portal blood supply cannot autoregulate. Thus, PPH-induced hypotension can cause severe ischemia (33).Currently, several factors have been described as contributing to the pathophysiology of Sheehan syndrome, including disseminated intravascular coagulation, autoimmune disorders, arterial spasm, hereditary factors, and arterial compression due to pituitary enlargement and/or a decrease in sella turcica volume (34). The incidence of Sheehan syndrome has reportedly declined dramatically in developed nations in recent years. This decline may be due to initiatives leading to improved delivery conditions, increased prenatal screening and monitoring for expectant mothers, and promoted evidence-based infant feeding practices. Nonetheless, hypopituitarism in developing and impoverished nations is still frequently caused by Sheehan syndrome (35).Insufficient blood flow to the pituitary gland due to diabetic microvascular lesions can potentially result in ischemic necrosis and hypopituitarism (36).In diabetic patients, hypopituitarism is also an uncommon cause of recurrent hypoglycemia,. Therefore, in order to diagnose and treat patients more effectively, doctors should completely understand the strong relationship between diabetes and hypopituitarism (37).

### 1.5 Infections, Infiltrative Lesions, and Toxin-Mediated Injuries

Infectious and infiltrative lesions, as well as toxin-mediated injury, are important causes of acquired hypopituitarism, which can impair pituitary function through direct tissue destruction, pituitary inflammation, or the influence of specific toxins (38).Granulomatous and infectious causes, In developed countries, tuberculosis as the cause of hypopituitarism is rare, and related reports are primarily case reports. At present, research suggests that tuberculosis is a mechanism leading to pituitary dysfunction, and tuberculosis can form space occupying lesions in the sellar region, which need to be differentiated from pituitary tumors and other tumors (39).In contrast, sarcoidosis is a more common granulomatous etiology that can involve the hypothalamus, pituitary stalk, or pituitary gland, leading to varying degrees of pituitary dysfunction and clinical manifestations of central diabetes insipidus (40).Viral infections, such as those caused by hantaviruses, can also cause hypopituitarism (41).Potential explanations for this phenomenon include bleeding during acute infection because of a marked drop in platelet count, which damages pituitary tissue, or post-infection hypotension, which results in an inadequate blood supply to the pituitary gland and ischemic necrosis of pituitary tissue (42).In addition to tuberculosis and Hantavirus, there are also syphilis (43), fungi (44), hemophilia (45), histiocytosis X (46), etc. These diseases have different mechanisms for causing pituitary dysfunction and/or central diabetes insipidus, so patients may present with mixed pituitary dysfunction and/or urinary incontinence. Toxin-mediated injuries are discussed primarily in snake bites, In Southeast Asian countries, snake bites are a rather prevalent cause of hypopituitarism, while not being a prominent cause in affluent nations (47).The pathogenesis of hypopituitarism following snake envenomation primarily involves pituitary hemorrhage and infarction, which are consequent to toxin-induced coagulopathy and direct vascular endothelial injury (48).According to recent research, it takes an average of 8.1 ± 3.6 years between a snake bite and an HP diagnosis (49).Nearly 30 years after Russell’s viper envenomation, a case report from 2022 detailed a diagnosis of panhypopituitarism and central diabetes insipidus; the 29-year delay in diagnosis may be the longest ever documented (50).Therefore, for all snake bite patients, routine and regular pituitary hormone screening and urine volume monitoring should be performed to prevent the occurrence of serious complications caused by delayed examinations (51).

### 1.6 Radiation damage

Radiotherapy is an important means of treating intracranial and skull base tumors, but pituitary dysfunction and diabetes insipidus are potential late complications of radiotherapy. Their pathogenesis involves direct damage to the level of the hypothalamus or pituitary gland, microvascular damage leading to ischemia, and the emergence of oxidative stress and inflammation (52).A key feature of radiation-induced hypopituitarism is its predictable sequence of onset. Because the growth hormone axis is the most susceptible to the neuroendocrine axis’s selective radiosensitivity, statistical data indicate that patients are more likely to experience growth hormone shortage following radiation therapy (53).This profile is evident in different patient groups; for example, a 2024 meta-analysis of adults receiving radiation therapy for brain, head and neck tumors reported a pooled prevalence of 61% for any pituitary insufficiency, with growth hormone deficiency (40%) being the most common, followed by gonadotropin (20%), adrenocorticotropic hormone (16%), and thyroid-stimulating hormone (16%) deficiencies (54).The same large cohort study of childhood brain tumor survivors treated with chemotherapy and radiation estimated the 40-year cumulative incidence of growth hormone deficiency, thyroid-stimulating hormone deficiency, adrenocorticotropic hormone deficiency, and gonadotropin deficiency to be 72.4%, 11.6%, 5.2%, and 24.4%, respectively (27.3 years of follow-up), highlighting the progressive nature and long-term risk of this complication (55)The risk and severity of hypopituitarism are related to many variables, such as the dose, type and location of radiation, as well as the age and gender of the patient. Radiation dose plays an important role in the treatment process. According to research, isolated growth hormone deficiency is associated with radiation exposure less than 30Gy. As the radiation dose increases to 30-50Gy, the incidence of growth hormone deficiency in patients increases dramatically and may reach 50%-100% (56).Radiation therapy techniques have been developed to mitigate these risks. Compared with traditional radiation therapy, stereotactic radiosurgery (SRS) has more accurate target positioning and dose delivery, thereby reducing the risk of adverse effects of radiation therapy. However, this risk advantage may be partly attributable to the shorter follow-up period of SRS compared with conventional radiation therapy (57).

### 1.7 Cranial brain injury or pituitary surgery

Damage to the brain brought on by a physical hit to the head is referred to as cranial brain injury. The consequences of traumatic brain injury on the neuroendocrine system have gradually come into focus since the first case of hypopituitarism linked to traumatic brain injury was documented in 1918 (58).The impact of traumatic brain injury on the neuroendocrine system has received increasing attention. The incidence of hypopituitarism after traumatic brain injury has been reported to vary significantly between studies (15-50%), and this variation can be attributed to heterogeneity of study populations, inconsistencies in diagnostic criteria, and differences in dynamic testing protocols (59).A 2015 study found that the combined prevalence of various pituitary abnormalities and post-TBI hypopituitarism is 4% and 15%, respectively. With a prevalence of 9%, growth hormone insufficiency is the most prevalent abnormality. Adrenocorticotropic hormone (ACTH) deficit comes in second with 6%, followed by LH/FSH deficiency with 5% and TSH deficiency with 1% (60).Notably, TBI severity assessed by the Glasgow Coma Scale (GCS) was poorly associated with the risk of hypopituitarism, suggesting that initial clinical symptoms are unreliable predictors of long-term endocrine sequelae (61).The pathophysiology of pituitary insufficiency after traumatic brain injury is thought to be multifaceted, ranging from primary mechanical injury to complex secondary insults. Besides, the development of TBI-induced hypopituitarism may potentially be linked to immunological processes and genetic predisposition. Subjects with the APOE3/E3 genotype had a substantially reduced incidence of pituitary dysfunction than subjects without this genotype (62).Long-term pituitary damage may also be significantly influenced by the development of high levels of APA/AHA (anti-pituitary antibodies) (62).According to research, about 16% of central diabetes insipidus is caused by damage to the hypothalamus, pituitary gland, or pituitary stalk (63).According to a literature report in 2022, a patient who suffered severe traumatic brain injury from a traffic accident subsequently developed permanent central diabetes insipidus (64).Although pituitary surgery has a therapeutic effect, it inherently carries the risk of iatrogenic damage to the pituitary axis. The mechanisms include direct cutting of thick functional pituitary tissue, surgical trauma caused by drilling or dissection, interruption of the pituitary stalk or its blood supply, postoperative scarring, and fibrosis (65).The most common postoperative endocrine sequelae are gonadotropin deficiency (2–15%) (66)and central diabetes insipidus, which is usually transient and occurs within hours to days after surgery (67).Therefore, active screening is recommended for high-risk groups, including patients with moderate to severe TBI, patients with recurrent head trauma, and all post-pituitary surgery patients. The diagnostic approach should include concurrent monitoring of anterior pituitary function and water balance.

### 1.8 Autoimmune

Autoimmunity is also an important cause of pituitary dysfunction and diabetes insipidus. Its pathological mechanism includes diffuse infiltration of immune cells (such as lymphocytes, plasma cells, etc.) in the pituitary gland. According to the etiology and pathological characteristics, autoimmune pituitary injury can be mainly divided into primary hypophysitis (such as lymphocytic hypophysitis, IgG4-related hypophysitis) and hypophysitis secondary to drugs such as immune checkpoint inhibitors (ICI) (68).A 2024 Mendelian bidirectional randomized study further deepened the causal relationship between immune cells and hypopituitarism (69).Among primary hypophysitis, the most common type of autoimmune hypophysitis is lymphocytic hypophysitis (70).Its epidemiological characteristics are distinct. The disease is more likely to occur in women, especially during pregnancy. Patients usually develop symptoms in late pregnancy or postpartum (71).Idiopathic diabetes insipidus accounts for about 30%. In clinical practice, when the specific cause cannot be determined, the possibility of autoimmune diabetes insipidus should be considered (72).With the booming development of tumor immunotherapy, the incidence of immune checkpoint inhibitor (ICI)-related hypophysitis has increased significantly (73).Its epidemiological characteristics vary depending on the type of drug, treatment regimen, and patient group. Relevant studies have shown that the overall incidence of pituitary inflammation induced by ICI is approximately 1.9%, and the risk of pituitary inflammation when using PD-1/PD-L1 inhibitors alone is relatively low (74).Anti-CTLA-4 drugs, such as ipilimumab, are associated with a higher risk of hypophysitis (75).In terms of population characteristics, ICI-related hypophysitis is more common in male patients and mostly occurs in middle-aged and elderly patients (76).The pathogenesis mainly involves immune reactivation, and the latest hypothesis suggests that CTLA-4 drugs may cause cross-reactivity between activated T cells and pituitary cells (77).The clinical manifestations of ICI-related hypophysitis are often insidious and non-specific. Most patients have ACTH deficiency as the first manifestation. Adrenocortical hormone (ACTH) deficiency leads to low cortisol levels, which can cause fatigue, hypotension and even shock (78).In terms of imaging, while some cases may show a transiently enlarged pituitary gland on MRI, a normal MRI does not rule out the diagnosis (79).It is recommended to establish a baseline pituitary function profile during treatment. It is worth noting that monitoring ACTH levels and their fluctuations may help predict the onset of ICI-related hypophysitis (80).According to relevant reports, in a few cases, the use of immune checkpoint inhibitors can also induce diabetes insipidus (81).Immunocheckpoint inhibitor discontinuation is still debatable in treatment due to the incidence of irAEs. Thus, future research on the therapeutic advantages and hazards of immunosuppressive treatment is required.

### 1.9 Empty sella syndrome

Empty sella turcica refers to a radiological sign in which the diaphragm of the sella is deficient, and the arachnoid membrane and cerebrospinal fluid herniate into the sella turcica, resulting in partial or partial filling of the sella turcica by cerebrospinal fluid and compression and flattening of the pituitary gland. It can be divided into two categories: primary and secondary (82).The most prevalent kind of empty sella syndrome is primary empty sella syndrome (PES), in which the sella turcica is free of pituitary abnormalities or other lesions that are obviously causally connected. Other conditions include pituitary tumors, intracranial or extracranial trauma, intracranial infection, or inflammation are frequently the cause of secondary empty sella syndrome (SES).According to some reports, between 8% and 35% of the general population has PES. The detection rate of PES has somewhat increased due to advancements in imaging technologies. Women make up the bulk of instances with empty sella syndrome, which peaks between the ages of 30 and 40 (83).Headache, visual field abnormalities, optic disc edema, gonadal dysfunction, hypothyroidism, and obesity are among the common clinical manifestations of PES. According to a recent multicenter retrospective cohort research, over 40% of PES patients had hypopituitarism. Interestingly, the study also discovered that male PES patients had a higher prevalence of hypopituitarism than female patients, indicating that gender may have an impact on the occurrence of PES with hypopituitarism. Future research on the connection between gender and PES hypopituitarism is required (84).In imaging, empty sella turcica can be divided into partial ES and complete ES according to the degree of filling of the sella turcica. When the bottom of the sella turcica is less than 50% full of serous or cerebrospinal fluid, it is referred to as partial ES. A condition known as complete ES occurs when the normal pituitary tissue is squashed into a thin sheet that is no thicker than 2 mm and the bottom of the sella turcica is filled with serous or cerebrospinal fluid to more than 50% of its capacity. Once diagnosed by imaging, clinical evaluation must be conducted to determine the presence of pituitary hormone deficiency. It should be noted that empty sella syndrome is usually less likely to cause diabetes insipidus. However, if patients exhibit symptoms of diabetes insipidus, the possibility of empty sella syndrome should be considered, whether it is partial or complete empty sella syndrome (85). A 2021 retrospective single-center study revealed the correlation between pituitary gland volume and clinical features: pituitary gland volume was negatively correlated with age and positively correlated with serum IGF-1 levels (83).According to the aforementioned analysis, more thorough research and volume measurements are required in the future to more precisely evaluate pituitary volume and its effects on human health, particularly with regard to the correlation between pituitary volume and patient clinical features.

### 1.10 COVID-19

Severe Acute Respiratory Syndrome Coronavirus 2 is called “Severe Acute Respiratory Syndrome Coronavirus 2” and is shortened to SARS-CoV-2.The pathogen responsible for COVID-19 (new coronavirus pneumonia) is this coronavirus. Through its surface spike protein, SARS-CoV-2 attaches itself to the angiotensin-converting enzyme 2 (ACE2) receptor on the surface of host cells. Proteases must further break the virus to liberate its internal genetic material in order to finish the infection process. In this procedure, serine protease 2 (TMPRSS2) is essential. The human body contains a large number of ACE2 receptors (86). Indeed, the pituitary and hypothalamus are thought to have comparatively high levels of ACE2 expression. As a result, a SARS-CoV-2 infection may impact other organs and systems in addition to the respiratory system, resulting in a number of serious illnesses (87).COVID-19 may have multiple effects on adenohypophyseal function. First, adenohypophyseal cells may sustain damage from direct viral invasion, which would impair their ability to secrete normally. Furthermore, pituitary gland tissue fibrosis and inflammatory damage may result from immunological and inflammatory reactions brought on by viruses. Hypopituitarism may result from several disease conditions, albeit the precise pathological process has not yet been thoroughly investigated. A 2022 study of 23 patients who died from COVID-19 confirmed the affinity of SARS-CoV-2 for the pituitary gland. The study found that the mRNA transcription levels of pituitary hormones and related regulatory genes were significantly down-regulated in the pituitary tissue of all patients (88).In 2022, a literature reported a case of central diabetes insipidus secondary to COVID-19 infection (89).The reciprocal link between COVID-19 and hypopituitarism has not yet been conclusively determined by current research. More research is required to fully understand this association, even though there is some evidence that hypopituitarism may be a result of COVID-19 interfering with pituitary function and that hypopituitarism may raise the risk and severity of COVID-19 infection (90).

### 1.11 Iron deposition

The aberrant buildup of iron in the pituitary gland, known as pituitary iron deposition, can impair pituitary function. Because iron is necessary for the production and release of pituitary hormones, the pituitary gland is extremely sensitive to iron metabolism. However, aberrant iron deposition in the pituitary gland may occur in some disease situations, such as hereditary hemochromatosis or disorders linked to high exogenous iron intake, such as hemochromatosis or blood transfusions, which can result in hypopituitarism (91).The core mechanism is that excessive reactive oxygen species (ROS) generated by iron catalysis induce mitochondrial dysfunction and lipid peroxidation, directly leading to apoptosis of pituitary secretory cells (92). The gonadotropin axis is usually the earliest and most susceptible, followed by the growth hormone axis (93). Due to repeated blood transfusions, thalassemia patients frequently experience iron overload, which can cause endocrine system malfunction. According to reports, between 25% and 33% of children with thalassemia may experience one or more endocrine issues. The risk of endocrine complications in adult patients is also greatly elevated, with estimates reaching 80% (94).Among these complications, pituitary dysfunction directly caused by iron deposition is particularly critical. It should be mentioned that thalassemia patients’ endocrine dysfunction brought on by iron excess is a complicated process, and further research is needed to determine the precise causes.

### 1.12 Others

In addition to the above reasons, malnutrition, neurological diseases, and idiopathic hypopituitarism are other causes of hypopituitarism. Patients with severe malnutrition will have changes in cerebral cortex and subcutaneous structures, which may be the structural basis of hypothalamic-pituitary axis dysfunction (95). Some neurological diseases have unique neurological manifestations worthy of attention (96). In addition, despite the continuous advancement of diagnostic technology, there is still a considerable proportion of hypopituitarism whose cause cannot be clearly determined and is classified as “idiopathic”. These factors are critical to a thorough understanding of the pathophysiology of hypopituitarism and are common in clinical practice. A comprehensive understanding of these causes is crucial for accurate identification and individualized treatment in clinical practice.

## 2 Summary

Congenital developmental defects, neoplastic lesions, infection and inflammation, autoimmune illnesses, trauma and surgery, and idiopathic causes are all part of the extremely diverse etiology of hypopituitarism. Our knowledge of the etiology and pathophysiology of hypopituitarism has advanced significantly with the ongoing development of contemporary medical technology, particularly the quick development of molecular biology, genetics, and imaging. Many key issues remain to be explored in depth. For example, the interaction between genetic factors and environmental factors, the precise role of autoimmunity in specific types of hypopituitarism, and how to diagnose and evaluate pituitary function earlier and more accurately are all difficult points that need to be overcome in future research. However, the results of earlier studies have greatly improved the diagnosis and treatment of hypopituitarism by offering a crucial theoretical foundation for clinical practice. Future research on the causes of hypopituitarism is expected to uncover additional pathogenic pathways, which will serve as a strong foundation for the creation of more precise and effective diagnostic techniques as well as therapeutic approaches. In addition to increasing the clinical cure rate for patients, this will encourage the broad use of customized medicine in the management of hypopituitarism, giving patients better access to healthcare and greatly enhancing their prognosis and quality of life.
